# Advances in Pathogenesis and Therapeutics of Hepatobiliary Diseases

**DOI:** 10.3390/biomedicines11041140

**Published:** 2023-04-10

**Authors:** Jing-Hua Wang

**Affiliations:** Liver and Immunology Research Center, Institute of Bioscience & Integrative Medicine, Daejeon University, 75, Daedeok-daero 176 Beon-Gil, Seo-gu, Dunsan-dong, Daejeon 35235, Republic of Korea; ewccwang@gmail.com; Tel.: +82-42-257-6397; Fax: +82-42-257-6398

The hepatobiliary system, comprising the liver, gallbladder, and bile ducts, performs a diverse array of functions that are essential to maintaining homeostasis [1], including digestion, nutrient metabolism, detoxification, coagulation, and immune modulation [2]. Alterations in the normal function of the hepatobiliary system can have serious implications for human health, resulting in a broad range of pathologies spanning from liver disease to bile system disorders.

The Special Issue of *Biomedicines*, entitled “Advances in Pathogenesis and Therapeutics of Hepatobiliary Diseases”, presents cutting-edge research findings, innovations, and insights into a wide range of topics related to the hepatobiliary system, such as the molecular mechanisms underlying disease pathogenesis, diagnostic advancements, novel therapeutic approaches, and prevention strategies. The Special Issue comprises seven original articles and four review articles regarding NAFLD, primary biliary cholangitis (PBC), hepatitis B and C, and hepatocellular carcinoma (HCC). 

NAFLD is currently a major global health issue. It affects approximately 25% of the global population [3], with the highest prevalence in Western countries (25–30%) [4]. It affects 80–100 million people in the United States, making it the most common type of chronic liver disease [3]. NAFLD is also prevalent in Asia, particularly in Korea (affecting up to 30.3% of adults; men: 41.1%, women: 20.3%) [5]. As a result, many researchers are working to identify methods to prevent and treat NAFLD [6,7,8,9]. However, the lack of FDA-approved therapeutic options for NAFLD remains a significant impediment, emphasizing the critical importance of timely and accurate diagnosis [10]. Herein, Cazac et al. offer a comprehensive review of ultrasound-based hepatic elastography as a non-invasive method for diagnosing NAFLD in patients with type 2 diabetes mellitus (T2DM) [11]. They also highlight the limitations of liver biopsy and the need for non-invasive diagnostic tools to assess NAFLD in high-risk patients [11]. Bertran et al. utilized systems biology tools and public databases to identify the potential molecular mechanisms linking RUNX1 and NAFLD, resulting in a promising therapeutic strategy and a novel approach to treating NAFLD [12]. Furthermore, Abu-Freha et al. shed light on an important and relatively under-researched field of study: sex-based differences in NAFLD [13]. They used clinical big data from Clalit Health Services (CHS) in Israel. This discovery would improve our understanding of sex-based differences in NAFLD by highlighting disparities in comorbidities, outcomes, and mortality rates between females and males [13].

Furthermore, cholestatic diseases are significant because they have the potential to cause severe liver damage and long-term complications, emphasizing the importance of early diagnosis and treatment. One review provided a comprehensive overview of cholestatic diseases, including subtypes and causes, as well as the most up-to-date treatment options for cholestatic diseases, including pharmacotherapeutic agents and gene therapy [14]. It is worth noting that the discussion of gene therapy for inherited and acquired cholestasis is insightful and demonstrates the future promise of using gene therapy to address this medical issue [14]. Another review summarized current knowledge regarding pharmacological interventions for the treatment of primary biliary cholangitis (PBC) [15]. As is known, ursodeoxycholic acid (UDCA) is the first-line therapy for PBC and has been proven to normalize liver markers, delay disease progression, and prolong transplant-free survival. Obeticholic acid (OCA) is the only approved second-line treatment for individuals with PBC who do not respond to UDCA. Floreani et al. provided a comprehensive review of the pharmacological properties of OCA, including its mechanism of action, as well as its tolerability and effectiveness in treating PBC patients [16]. Moreover, one cohort study conducted by Bauer and colleagues, comprising 138 PBC patients and 90 non-PBC patients, revealed that the presence of antibodies against the kelch-like 12 (KLHL12) protein is a highly specific marker for diagnosing PBC [17]. When used in conjunction with other markers, it has the potential to significantly enhance the accuracy of PBC diagnosis.

Furthermore, hepatitis B and C also represent major global health issues. According to the World Health Organization (WHO), an estimated 354 million people worldwide are living with viral hepatitis B and C [18]. Infection with HBV and HCV can increase the risk of developing liver fibrosis, cirrhosis, and even liver cancer [19]. Three reports are presented here that are associated with the diagnosis, treatment, and pathogenesis of hepatitis induced by HBV/HCV. A study conducted in Spain found variants with indels in the 3’ end of HBX in most of their chronic hepatitis B (CHB) patients [20]. These variants encoded alternative versions of HBx that have the potential to play a functional role and/or alter transcriptional regulation [20]. This will provide useful insight into the genetic variability of HBV in CHB patients who do not have HCC. Another study investigated the correlation between serum miR-125b levels and liver fibrosis in CHB patients after 12 months of nucleoside analog treatment [21]. The results indicated that there is an inverse relationship between miR-125b levels and the post-treatment FIB-4 index score, but it is not a significant predictor of a higher score. Age, baseline platelet count, and ALT level were all independent predictors of a FIB-index greater than 2.9 post-treatment [21]. The last study investigated the long-term effects of direct-acting antiviral (DAA) regimens on mitochondrial DNA (mtDNA) instability in people who inject drugs (PWID) with chronic HCV [22]. The mtDNA parameters were measured nine months after treatment, and the percentage of deleted mtDNA genomes increased over time due to their replicative advantage over elimination processes [22].

In terms of clinical therapy for HCC, Tay et al. compared the clinical outcomes of trans-arterial chemoembolization (RFA) and radiofrequency ablation (TACE) as initial monotherapy for patients with early-stage HCC [23]. According to their findings, TACE could be considered a potential treatment option for patients who are unsuitable candidates for other therapies [23].

Overall, I anticipate that the collection of articles in this Special Issue will contribute significantly to ongoing efforts to improve the diagnosis, prevention, and treatment of hepatobiliary diseases, resulting in better patient outcomes and overall public health.
